# Serine Protease Autotransporters of *Enterobacteriaceae* (SPATEs): Biogenesis and Function

**DOI:** 10.3390/toxins2061179

**Published:** 2010-05-28

**Authors:** Nathalie Dautin

**Affiliations:** Department of Biology, The Catholic University of America, 620 Michigan Avenue N.E., Washington, DC, 20064, USA; Email: dautin@cua.edu; Tel.: +1-202-319-5278; Fax: +1-202-319-5721

**Keywords:** SPATE, autotransporters, pathogenic *E. coli*, *Shigella*, EspP, Pet, EspC, Sat, Vat, Hbp, EpeA, Pic, SepA, SigA, Tsh

## Abstract

Serine Protease Autotransporters of *Enterobacteriaceae* (SPATEs) constitute a large family of proteases secreted by *Escherichia coli* and *Shigella*. SPATEs exhibit two distinct proteolytic activities. First, a C-terminal catalytic site triggers an intra-molecular cleavage that releases the N-terminal portion of these proteins in the extracellular medium. Second, the secreted N-terminal domains of SPATEs are themselves proteases; each contains a canonical serine-protease catalytic site. Some of these secreted proteases are toxins, eliciting various effects on mammalian cells. Here, we discuss the biogenesis of SPATEs and their function as toxins.

## 1. Introduction

### 1.1. The Autotransporter Pathway

**Figure 1:**
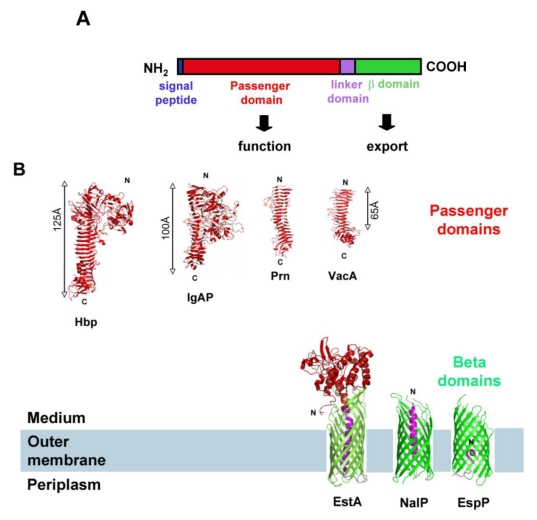
Autotransporter Proteins: Common organization and structure (**A**). Domain organization of AT proteins. (**B**). Crystallographic structure of representative AT domains. Passenger domains are shown in red. Hbp: Hemoglobin protease from *E. coli* (PDB entry 1WXR) [5], Prn: *Bordetella pertussis* Pertactin (PDB entry 1DAB) [4], VacA: *Helicobacter pylori* vacuolating toxin fragment p55 (PDB entry 2QV3) [7], and IgAP: *Haemophilus influenzae* immunoglobulin A1 protease (PDB entry 3H09) [8]. β-domains are shown in green: EspP from *E. coli* O157:H7 (PDB entry 2QOM) [11], NalP from *Neisseria meningitidis* (PDB entry 1UYN) [10], EstA from *Pseudomonas aeruginosa* (PDB entry 3KVN) [9]. Linker domains are shown in magenta.

SPATE (Serine Protease Autotransporters of *Enterobactericeae*) is a family of extracellular proteases produced by the *Enterobacteriaceae*. As their name indicates, they are “autotransporters” (ATs), meaning they are secreted by the Type Va secretion system from gram-negative bacteria. ATs are very diverse in their function (adhesin, protease, esterase, lipase, *etc.*) [1], but it is assumed that they share the same export mechanism. They are recognized based on their common organization: they are comprised of an N-terminal, *sec*-dependent, signal-peptide required for targeting to- and export through the inner membrane, followed by a “passenger domain” (which is the functional, secreted part of the protein), and a C-terminal β-domain (or ‘translocator” domain), which folds as a β-barrel in the outer membrane (OM). The C-terminal β-domain is necessary for translocation of the passenger domain through the OM [2,3]. The region linking the passenger domain and the β-barrel is referred to as the “linker domain” (Figure 1) [2,3].

Despite their diversity in function, most autotransporter passenger domains fold or are predicted to fold as β-helices [4,5,6,7,8]. Usually, the β-helix forms a “spine” to which additional, functional domains are attached. However, exceptions exist: the recently crystallized EstA autotransporter from *Pseudomonas aeruginosa* harbors a globular, α-helical passenger domain, with no β-helix domain (Figure 1) [9]. 

Autotransporter beta-domains are structurally very conserved. The three AT β-domains crystallized to date form 12-stranded β-barrels that are almost perfectly superimposable [9,10,11]. Approximately 30 amino acid residues, located upstream from the β-barrel-forming region in the primary sequence, form an α-helix in the pore of the β-barrels and connect the N-terminus of the β-barrel with the extracellular passenger domain (Figure 1). This region, also called the “linker domain” is necessary for the folding and stability of the β-barrel [10,12,13]. 

Although the domain organization and, to a certain degree, the three-dimensional structure of autotransporters is conserved, their sequences show only weak homology. However, AT subfamilies can be identified based upon increased amino-acid sequence identity. One of them is the SPATE subfamily, initially identified by Henderson *et al*. [2].

### 1.2. The SPATE Subfamily of Autotransporters

The SPATE subfamily of autotransporters is currently -composed of: EspP (extracellular serine protease plasmid (pO157)-encoded), initially designated PssA (protease secreted by STEC), and EpeA (EHEC plasmid-encoded autotransporter) from enterohaemorrhagic *E. coli* (EHEC) [14,15,16], Pet (plasmid-encoded toxin) from enteroaggregative *E. coli* (EAEC) [17], Pic (protease involved in intestinal colonization) from EAEC, uropathogenic *E. coli* (UPEC) and *Shigella* [18,19], EspC (EPEC secreted protein C) and Hbp (hemoglobin protease or hemoglobin binding protein) from enteropathogenic *E. coli* (EPEC) [20,21], Sat (secreted autotransporter toxin) from UPEC [22], Tsh (temperature-sensitive hemagglutinin) and Vat (vacuolating autotransporter toxin) from avian pathogenic *E. coli* (APEC) [23,24], EatA (ETEC autotransporter A) from enterotoxinogenic *E. coli* (ETEC) [25], EspI (*E.coli* secreted protease, island-encoded) from Shiga toxin producing *E. coli* (STEC) [26], EaaA and EaaC from the non-pathogenic ECOR-9 *E. coli* strain [27]; as well as ATs from *Shigella flexneri*: SepA (*Shigella* extracellular protein A) [28], SigA [29], and one protein from *Salmonella bongori:* Boa (*bongori* autotransporter) [30]. Yen *et al*. also reported the identification of additional SPATEs in *Citrobacter* and *E. coli* strains E22, B7A, and F11 [31].

Proteins belonging to the SPATE family display the typical characteristics of autotransporters: they are composed of a signal sequence, a passenger domain secreted in the extracellular medium, and a C-terminal β-domain necessary for translocation of the passenger domain through the outer membrane. The crystal structure of one SPATE passenger domain has been solved (Hbp), as well as that of one SPATE β-domain (EspP) [5,11]. Like for other ATs, the EspP β-domain folds as a 12-stranded β-barrel, whereas the Hbp passenger domain forms a β−helix to which a protease domain is attached (Figure 1).

SPATEs have been grouped in a family based on several criteria:

(1) They display a very conserved β-domain. In pair-wise comparison, sequence identity among SPATE proteins ranges from 25 to 55%. However, the conserved residues are not equally spread over the length of the proteins. The passenger domains are variable in length (between 954 and 1050 residues) and show between 23 and 50% amino-acid identity. In contrast, the β-domains are all exactly 277-residues long and 60 to 99% identical. A few SPATEs are more conserved: EaaA and EaaC, as well as Tsh and Hbp are almost identical (EaaA and EaaC differ by eight residues, Tsh and Hbp differ by only two). In addition, Vat and Tsh/Hbp are 77.5% identical and SepA and EatA are 72.8% identical. In these pairs, both the β-domains and the passenger domains are conserved, with most of the divergence occurring at the N-terminus of the protein. 

(2) As their name specifies, SPATEs are proteases. They all ccontain a conserved serine-protease motif (GD*S*GS where *S* is the catalytic serine) at the N-terminus of their passenger domain (between residues 250–270). 

(3) Whereas some autotransporters remain intact in the outer membrane after secretion, others are cleaved between the passenger domain and the β-domain, thus releasing the passenger domain into the extracellular milieu. All SPATEs are cleaved between the passenger domain and the β-domain after translocation of their passenger domain through the outer membrane. The cleavage occurs at a conserved FxxEVNNLNK site located in the linker domain, with the processing always occurring between the two asparagines. Various mechanisms have been involved in autotransporter cleavage [3]. In the case of SPATE proteins, the processing is thought to be autoproteolytic, intra-molecular, and catalyzed by the β-domain (see below).

(4) All SPATE proteins have unusually long (>49 amino-acids) signal sequences. This type of signal sequence is found in ~10% of autotransporters, as well as in proteins secreted by the “two-partner secretion system” (or type Vb secretion system). 

(5) SPATEs are usually among the most abundant proteins secreted by the parental strain, at least in laboratory conditions.

## 2. SPATEs Genes: Location and Evolution

SPATE-encoding genes are located on mobile genetic elements, such as plasmids (EspP [14,15,16], Pet [17], EatA [25], EpeA [16], Hbp [5], SepA [28], Tsh [32]); prophages (EaaA and EaaC [27]), or chromosomal pathogenicity islands (Sat [22], EspI [26], SigA [29], EspC [33], Pic [19,34], Vat [35]). Most of these genes are flanked by IS-like elements and have GC% significantly different from the genome of their producing strain [2,15,17,21,22,32,33,35]. Altogether, this suggests that SPATE genes have been acquired by horizontal gene transfers. Horizontal gene transfers have been reported for non-SPATE autotransporter genes [36], and have been proposed in the case of the SPATE Boa, which is only detected in a single species of *Salmonella* [31]. In addition, a SPATE gene (*eaaC*) located on a prophage, could be experimentally transferred between two *E. coli* strains by transduction [27]. Still, the phylogenic clustering of AT proteins also suggests that if horizontal transfer events occurred, they were rare and limited to phylogenetically related species. Yen *et al*. proposed that most ATs instead evolved through speciation and duplication events rather than horizontal gene transfer [37]. The presence of the two identical genes *eaaA* and *eaaC* in the ECOR-9 strain of *E. coli* indeed suggests that duplication of SPATE genes occurred [27].

In addition, the high conservation of the β-domain sequence compared with the passenger domain sequence, suggests that ATs might have arisen from the fusion between a common, generic translocator domain and various functionally divergent passenger domains [38]. This hypothesis is supported in the SPATE family where the β-domains are much more conserved than the passenger domains. Several members actually share almost identical β-domains but divergent passenger domains (for example, EspI, EspP, and EpeA β-domains are >95% identical, whereas their passenger domains are less than 35% identical; Pet and EaaA/EaaC β-domains are >90% identical whereas their passenger domains are <25% identical). After fusion to the translocator domain, the passenger domains might also have evolved independently even further, through point mutations and recombination events. For example, the gene encoding EspP displays a genetic heterogeneity that led to the identification of four different alleles [39]. This heterogeneity is due to point mutations, as well as recombination events. In particular, two of these alleles acquired a DNA fragment from *espI*, another SPATE-encoding gene [39], which indicates that recombination between SPATE genes occurred. These events have a reasonable chance of taking place; some strains possess several SPATE genes (for example the *E. coli* O113:H21 strain EH41 possess *epeA* and *espP* on a plasmid and *espI* on the chromosome [16], and UPEC CFT073 encodes Sat, Pic and Vat [19]). Recombination events between SPATE passenger domains are also suggested by phylogenetic studies [31,40]. Ultimately, this complex evolution would have led to the extreme functional diversity observed within autotransporters in general, but also in the more closely related SPATEs. 

## 3. Regulation of Expression

The exact mechanism by which SPATE expression is regulated has not been studied in much detail. Their expression seems to be regulated by environmental stimuli (temperature, oxygen availability, pH) as well as by quorum sensing. In contrast with Pet, for which expression does not seem to be affected by temperature [17], SigA, Pic, EpeA, EspP, EspC, Tsh, and SepA are all thermoregulated, showing maximal expression at 37 °C compared with lower (20–30 °C) or higher (40–42 °C) temperatures [14,16,18,23,28,29,35,41,42]. Such thermoregulation is often observed for virulence factors that need only to be expressed in the host in the context of infection but that are not necessary for the bacterium to survive in the environment. The expression of several SPATEs in the context of infection has actually been confirmed by the detection of anti-SPATE antibodies in the serum of infected patients [14,15,17,43,44,45]. Immune response to SPATEs was also observed in animal models of infection for Sat and SepA [22,28]. The mechanism or pathways involved in SPATE thermoregulation are unknown. Expression of some SPATEs is also pH-dependent, with optimum expression at alkaline pH (pH 7.0–9.0 for EspP [14], pH 8.0 for EpeA [16], pH 9.0 for Pic [18]). Hbp production was enhanced under anaerobic conditions [5], whereas osmolarity had no effect on Pic or Tsh expression [18,42]. Interestingly, EspC expression, in addition to being regulated by temperature, is also dependent upon the medium composition: the protein is expressed in tissue culture medium such as MEM medium, but not in LB or M9 medium. Additionally, EspC expression increases upon contact with host cells [20,41]. *espC* transcription is also regulated by quorum sensing, through the Per/Ler regulatory pathway in EPEC. Although Ler is also present in EHEC, it does not influence EspP expression [46]. 

## 4. Biogenesis

Autotransporters, including SPATE proteins, are transported through the inner membrane via the Sec-translocon. Once in the periplasm, the C-terminal domain folds as a β-barrel in the outer membrane, forming a pore. The passenger domain is then translocated through the outer membrane, cleaved from the β-barrel and released in the extracellular medium, where it will fulfill its function, usually related to the virulence of the producing strain. 

### 4.1. Export through the Inner Membrane

After synthesis, SPATE proteins are targeted to the Sec-translocon which catalyses their transport through the inner-membrane. To be targeted to the Sec-translocon, presecretory proteins need to harbor a N-terminal, cleavable, “signal sequence”. These sequences are usually ~20–30 residues long, highly variable in sequence and organized in three domains: a N-terminal, relatively basic “N-domain”, a “H-domain” rich in hydrophobic residues, and a “C-domain” where the cleavage by signal peptidase occurs. In the case of SPATE proteins, the signal sequence is at least 49 residues long and is comprised of a ~25 residues N-terminal conserved extension and a C-terminal non-conserved region, which resembles a conventional signal peptide (Table 1). The origin of the N-terminal extension is unknown, it is found in ~10% of all autotransporters, but in 100% of SPATEs [3]. It is highly conserved, with an amino-acid sequence identity ranging between 37.5% and 96% in the SPATE family. An increase in identity in the N-terminal extension does not correlate with an increase in β-domain or passenger domain amino-acid identity, suggesting that this extension was acquired independently from either of these domains.

**Table 1:** Sequence comparison of SPATEs signal sequences. Residues strictly conserved are highlighted in red, residues most frequently found at each position are highlighted in blue and conservative mutations are highlighted in grey. The cleavage site for signal peptidase is indicated by the symbol <>.

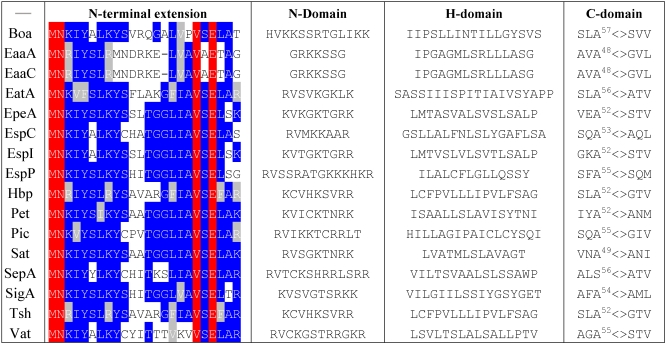

The C-terminal “classical” signal peptide is necessary and sufficient for inner membrane translocation. In contrast, the N-terminal extension is not a targeting signal by itself, and is not necessary for secretion through the inner membrane [47,48,49,50]. However, this sequence influences the targeting pathway to the inner membrane. Secreted proteins can be targeted to the Sec-translocon either via the SRP (signal recognition particle) pathway, or through a SecB-dependent pathway. The SRP ribonucleoprotein complex promotes cotranslational targeting of protein by interacting with highly hydrophobic signal sequences when they emerge from the ribosome. In the SecB-dependent pathway, presecretory proteins are targeted to the inner membrane post-translationally by a mechanism that requires the SecB chaperone to keep the protein in a secretion-competent state within the cytoplasm. Both EspP and Pet are secreted post-translationally, in a SecB-dependent manner. However, the deletion of the N-terminal extension increases the Pet rate of inner membrane secretion and reroutes EspP to the SRP pathway [48,49]. This suggests that the N-terminal extension may either bind to an unknown cytoplasmic factor that delays inner membrane translocation, or prevents SRP from binding to the C-terminal region of the signal peptide [48,49]. In contrast to EspP and Pet, Hbp is targeted to the inner membrane cotranslationally, in a SRP-dependent fashion [50,51]. The difference in targeting observed between these SPATE proteins might be explained by the increased hydrophobicity of the Hbp H-domain compared with EspP and Pet [50]. Since SRP has higher affinity for hydrophobic signal sequences, it is possible that the higher hydrophobicity of this particular signal peptide overcomes the inhibitory effect of the N-terminal extension. However, increasing the hydrophobicity of EspP H-domain by site-directed mutagenesis did not influence the targeting pathway of this SPATE [48]. Another possibility might be that small amino-acid variations, in the N-terminal extension itself, affect the ability of this sequence to inhibit SRP binding. It is already known that mutation of the conserved Asn in position 2 of the extension is sufficient to abolish SRP-binding inhibition and reroute EspP to the SRP pathway [48]. However, other positions might also be important in inhibiting SRP binding. In this regard, it is interesting to note that EspP and Pet extensions have higher identity to each other than to the Hbp extension (Table 1). Thus, it might be interesting to test whether exchanging Hbp and EspP/Pet N-terminal extensions affect SRP binding or the targeting pathway of these proteins to the inner membrane, or both.

### 4.2. Transport through the Periplasm

In addition to a role in protein targeting, it was also proposed that the conserved N-terminal extension could affect later steps in biogenesis. Indeed, despite the fact that the signal sequence is cleaved when the protein crosses the inner membrane, Szabadi *et al*. showed that deleting the N-terminal extension severely impairs EspP transport through the outer membrane [47]. To explain this observation, they proposed a model in which the N-terminal extension delays EspP release in the periplasm via a prolonged interaction with the Sec-translocon. This delay would in turn prevent misfolding of the protein in the periplasm, and allow proper transport through the outer membrane. In contrast however, deletion of the N-terminal extension did not affect Hbp outer membrane translocation, since the protein was released normally in the extracellular medium [50]. Whether the disparity in results obtained between EspP and Hbp are due to variations in experimental conditions or intrinsic differences between Hbp and EspP (such as folding rate of the passenger domain or ability of the extension to tether the proteins to the Sec-translocon) is currently unknown. In any case, if this extension is indeed important for autotransporter biogenesis, one might ask why only ~10% of autotransporters harbor this sequence. It is possible that some autotransporters (especially SPATEs), are particularly prone to aggregation in the periplasm and thus need this sequence for secretion through the envelope to occur. ATs lacking this sequence might be less prone to aggregation or have evolved other mechanisms to prevent misfolding in the periplasm. Such mechanisms could involve a reduced folding rate or interaction with specific periplasmic chaperones. The passenger domain of pertactin, a non-SPATE AT that does not possess a N-terminal extension, indeed folds very slowly *in vitro*, without forming aggregates [52]. However, this does not appear to be specific to AT missing the N-terminal extension, since the folding of the SPATE Pet was not significantly different from Pertactin *in vitro* [53]. *In vitro* folding is nevertheless very different from *in vivo* events, where secreted proteins might encounter chaperones in the periplasm. Indeed, two SPATE “stalled” mutants have recently been used to identify potential partners of SPATE proteins during secretion. Using this strategy, Hbp was found to interact with the periplasmic chaperone SurA, whereas EspP passenger domain cross-linked with both SurA and Skp [54,55]. Although direct interactions between the wild-type SPATEs and the respective chaperones were not observed in these studies, wild-type denatured EspP was shown to interact with SurA, Skp, and DegP in surface plasmon resonance and two-hybrid assays [56]. In addition, experiments with *E. coli* mutant strains further suggest that these periplasmic chaperones are important for SPATE secretion. Indeed, EspP secretion was reduced in *surA*, *skp*, or *degP* mutants of *E. coli*, and Hbp secretion was affected in a *surA* strain [54,56]. Interestingly, *surA* deletion had a much more pronounced effect on Hbp biogenesis than with EspP. In a *surA* strain, EspP is translocated through the OM and cleaved, but the level of cleaved EspP passenger domain found in the extracellular medium was slightly reduced compared with the level found in a wild-type *E. coli* strain [56]. Hbp, in contrast, is unprocessed in a *surA E. coli* strain [54]. Hbp secretion through the outer membrane is not affected by the absence of the N-terminal extension, which suggests (according to the model proposed by Szabadi *et al*.) that Hbp extension does not tether the protein to the inner membrane like it apparently does for EspP. In the absence of such tethering, Hbp might be more dependent on chaperone like SurA to avoid misfolding in the periplasm, which would explain the more pronounced effect of *surA* mutation on Hbp biogenesis compared with that of EspP. Thus, SPATEs apparently need to be kept in a secretion-competent conformation in order to be secreted properly through the outer membrane. This is apparently possible either through a prolonged interaction with the Sec-translocon or via interaction with periplasmic chaperones, or both. The question of what exactly is a “secretion-competent” state for autotransporters is still a matter of debate (see below).

### 4.3. Transport through the Outer Membrane

Once in the periplasm, the autotransporter β domain is inserted into the outer membrane (OM), where it forms a β-barrel. The passenger domain is then transported to the extracellular medium. Deletion of an AT β domain abolishes the translocation of the passenger domain through the OM; this domain is thus essential to the translocation. However, its exact role is still unknown. Because β-barrels can form pores in the OM, it was initially proposed that AT passenger domains are translocated through the OM via the pore formed by their associated β-barrel (hence the name “autotransporters”) [57]. Accordingly, the crystallographic structures of the 3 AT β−domains solved to date (Figure 1) showed that the C-terminus of the passenger domain resides in the center of the pore formed by the β domain [9,10,11]. These crystallographic structures also showed that the pores of these barrels are too narrow (~10 Å) to translocate folded passenger domains [9,10,11]. This is inconsistent with numerous studies reporting the translocation, by ATs, of folded passenger domains through the OM. As a consequence, the idea of ATs mediating their own transport through the OM has been challenged. Today, three models have been proposed to explain translocation of AT passenger domains through the OM [3,58].

(1) In the “hairpin” model, the passenger domain is transported through the pore of the β-barrel, with the C-terminus of the passenger first. This model implies that the passenger is unfolded when it crosses the OM.

(2) A study with IgA protease, an AT from *Neisseria*, showed that this particular AT is able to form multimers in the OM, which led to a second model in which the passenger domain translocation would occur through a central channel formed by the walls of 6 or more β-barrels [59]. Because the pore formed by these oligomers would be wider than the pore formed by an individual AT β-barrel, it would be able to translocate folded domains across the OM.

(3) Finally, a third model proposes that AT are inserted in the OM via the general OM-protein assembly machinery Omp85/YaeT [10,58,61], and that the passenger domain translocation is concomitant to the insertion of the AT in the OM. 

SPATEs β-domains for which information on oligomerization states is available (EspP and Tsh) were found to be monomeric [11,61,62]. These studies suggest that the translocation of SPATE passenger domains through the OM does not occur via an oligomeric pore formed by their β domains. In addition, folding of a heterologous passenger domain (cholera toxin B) in the periplasm does not prevent its translocation across the OM by the EspP β-domain. This argues against the hairpin model [62]. In contrast, Jong *et al*. found that although the Hbp passenger domain folds to a certain extend in the periplasm, tightly folded domains attached to this passenger can block translocation [63]. Finally, recent studies found that EspP and Hbp both interact with Omp85/YaeT during secretion, and that depletion of Omp85 strongly affects the secretion of Hbp through the bacterial envelop [54,55,56]. Altogether, these results suggest that SPATEs transport their passenger domain through the OM via a mechanism depending on both their own β-domains and Omp85. The precise mechanism of this translocation however, is still unknown.

### 4.4. Cleavage

**Figure 2:**
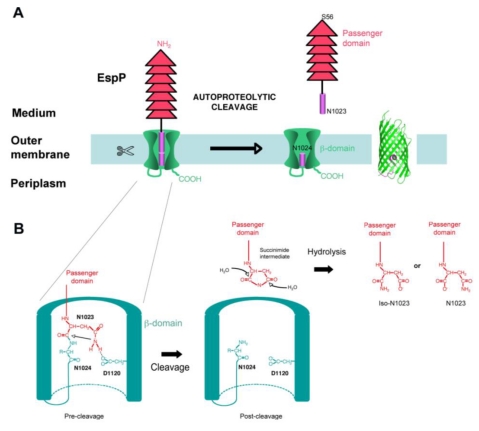
Proposed mechanism of SPATEs autoproteolytic processing. (**A**). EspP was proposed to be cleaved by an autoproteolytic mechanism taking place in the pore of the autotransporter β-barrel. (**B**). After translocation of the passenger domain across the OM, Asn1023 is positioned close to Asp1120. As a consequence of hydrogen bonding between the two side chains, Asn1023 amide group performs a nucleophilic attack on the Asn1023–Asn1024 peptide bond. This reaction results in Asn1023 cyclization and the production of a succinimide intermediate at the C-terminus of the passenger domain, which will be ultimately hydrolyzed into a mixture of asparagine and iso-asparagine [64,65].

Whereas some autotransporters remain intact in the outer membrane after secretion (e.g., EstA, Figure 1), others are cleaved between the passenger domain and the β-domain, thus releasing the passenger domain in the extracellular milieu. Cleavage of the passenger domain from the β-domain can occur by various mechanisms. Certain ATs are cleaved autoproteolytically, with a protease domain located in the passenger domain being necessary for processing. One AT (IcsA from *Shigella flexneri*), is cleaved by an outer membrane protease homologous to OmpT (IcsP), and some autotransporters are cleaved by other ATs (for example, NalP cleaves IgA-P) [3]. All SPATEs are cleaved, after translocation of their passenger domain through the outer membrane, between the passenger domain and the β-domain. The cleavage occurs at a conserved FxxEVNNLNK site located in the linker domain, with the processing always occurring between the 2 asparagines [17,18,42]. Very early on, it was shown that the conserved serine protease site located in the first third of the passenger domain was not involved in the processing of the passenger domain from the β-domain [14,20,25,26,28,42,47]. In addition, expression of SPATEs in strains deleted of outer membrane or periplasmic proteases such as OmpT, OmpP, or DegP, did not affect cleavage either [14,17,28,42]. Recently, we found that the conserved cleavage site (EVNNLN) is actually located in the pore of EspP β-barrel after translocation, suggesting that it might not be accessible to any periplasmic or outer membrane proteases. As a consequence, we tested the hypothesis that the SPATE β-barrel could be itself the protease responsible for cleavage. Indeed, we found that purified EspP was still processed *in vitro* and that mutations in EspP β-barrel abolish cleavage. Specifically, we identified two positions that are crucial for cleavage: Asp1120, which is located in the β-domain and is facing the pore of the barrel, and Asn1023, which is the residue just upstream from the cleavage site [64]. The implication of one aspartate and one asparagine in an autoproteolytic reaction, with the Asn at the P1 residue of the cleavage site, was highly reminiscent of the autoproteolytic reaction proposed for the capsid protein of Nodavirus [65]. As a consequence, we proposed that EspP is cleaved by a similar autoproteolytic mechanism, with the cyclization of the Asn located at position P1 of the cleavage site being catalyzed by the Asp in position 1120 of the β-barrel (Figure 2) [64]. 

In such a mechanism, the cyclization of the catalytic Asn results in the formation of a succinimide intermediate, which is then hydrolyzed into either asparagine or iso-asparagine [65]. Accordingly, ,analysis of the secreted EspP passenger domain by mass spectrometry showed that the passenger domain is secreted in two forms: one being 18 Da smaller than the theoretical mass of the domain and probably corresponds to the succinimide intermediate, and one form corresponding to the passenger domain with either an asparagine or iso-asparagine at the C-terminus [64]. We also found a mixture of Asn and Iso-Asn at the C-terminus of the secreted EspP passenger domain [64]. Although outer membrane proteases are well documented (referred to as “omptins”), this is the first example of an OM protease that possesses a catalytic site inside the pore of the barrel. Omptins’ catalytic residues, in contrast, are located in the extracellular loops of the protein. The SPATE intra-barrel cleavage was further confirmed by the resolution of EspP β-barrel structure (post-cleavage), which clearly shows a fragment going half-way through the pore of the barrel, instead of an α-helix reaching the extracellular side of the membrane, which is seen in ATs for which cleavage takes place in the extracellular medium (Figure 1) [11]. 

The residues important for cleavage are conserved in all SPATEs, suggesting that all members of this family are processed by the same autoproteolytic mechanism. Although this has not been confirmed, Kostakioti *et al*., found that, like in EspP, mutation of the Asn at position P1 of the cleavage site abolishes cleavage in Tsh [67]. In addition, several mutations located in the linker domain also affect cleavage in both EspP and Tsh [64,66]. However, some of these mutations also affect passenger domain translocation in Tsh, whereas they did not in EspP [64,66]. Interestingly, we also found that this mechanism is conserved in certain non-SPATE autotransporters, such as the autotransporters BrkA and Pertactin from *Bordetella pertussis* [64]. The cleavage of SPATEs is very fast, with most of the protein being cleaved in only few minutes [47]. The passenger domain is also quickly released into the medium, but some cleaved passenger domain also remains associated with cells for an undetermined time [47]. Considering the variety of cleavage mechanisms found among ATs, why SPATEs have evolved an intra-barrel mechanism is puzzling, especially considering the fact that they possess a proteolytic activity in their passenger domain that they could potentially use to separate the passenger from the β-domain, in a mechanism similar to IgAP. This mechanism is probably not just a consequence of the fusion of the generic β-barrel, since it is also found in some *Bordetella* ATs, which have very low homology with SPATE β-domains. In theory, intramolecular cleavage would be faster than intermolecular cleavage. Thus, this intramolecular cleavage mechanism may have been favored in ATs for kinetic reasons. However, no comparison of cleavage rate among ATs has been performed yet. Also, whether an intramolecular cleavage catalyzed by the β-domain is faster than one catalyzed by the passenger domain will depend mainly on the folding state of the passenger domain during secretion. Indeed, cleavage in SPATEs occurs immediately after passenger translocation is completed and is probably not dependent upon passenger domain folding. In contrast, cleavage by the passenger domain requires the passenger domain (or at least domain 1) to be folded in the extracellular space. If this domain is not folded during translocation, then cleavage would have to “wait” for folding of the proteolytic domain. Considering the slow folding rate of the autotransporter *in vitro*, this might considerably delay cleavage [52]. Thus, the diversity in cleavage mechanisms among ATs might lead to differences in cleavage rates or cleavage efficiency. However, this explanation needs to be tested experimentally. Whether such differences would be important for the function of these proteins is not known. It is not known either if the cleavage itself is important for the virulence function of the SPATEs. It is probable that SPATEs need to be released from the cells (and thus cleaved) to reach their host target, but this has not been clearly established. The absence of adequate animal models for most of the SPATE producing strains precludes the comparison of pathogenesis between a strain expressing a wild-type SPATE versus one expressing an uncleaved SPATE mutant.

## 5. Function

SPATEs were initially identified as proteins secreted by bacterial species that are pathogenic to humans or animals (diarrheagenic *E. coli* and *Shigella*). Although SPATE genes have now also been detected in non-pathogenic strains of *E. coli* [27], they seem to be more strongly associated with pathogenic strains [30,67]. As a consequence, numerous studies have been performed in order to identify their potential role in pathogenesis. However, most SPATEs are produced by pathogenic species for which relevant animal models of infection are lacking, thus making a clear determination of their contribution to disease difficult. Still, the study of these proteins *in vitro* has provided valuable information about their putative role in pathogenesis.

### 5.1. Proteolytic Activity

As previously mentioned, all SPATEs possess a serine protease motif in the first third of their passenger domains. The actual proteolytic activity of this domain has been demonstrated for most of the family members (exceptions are EaaA, EaaC and Boa, for which protein expression itself has not been verified yet). SPATEs are indeed able to cleave various substrate proteins *in vitro* (Table 2). In addition, their membership in the serine protease clan was demonstrated by the use of specific serine protease inhibitors and site-directed mutagenesis of the putative catalytic serine present in the motif GD*S*GS. Both actions abolished proteolytic activity. Serine proteases rely on a catalytic triad for function. In EspP, the two additional catalytic residues were identified by site-directed mutagenesis as Asp156 and His127 [68]. These two amino-acids are, like the serine present in the GDSGS motif, conserved among SPATEs, despite low sequence identity in the protease domain. Hbp passenger domain structure has been solved (Figure 1, [5]) and shows that the protease domain (also called “domain 1”) forms an independent globular domain attached to the β-helical “spine” of the passenger (Figure 1). Although the proteolytic activity of the isolated domain 1 has not been tested, it seems that only this domain is necessary for proteolytic activity. Indeed, mutations abolishing proteolytic activity were only identified in this region [68]. This domain adopts a typical chymotrypsin-like fold, with the three catalytic residues (Ser207, Asp101 and His73 in Hbp) located in close proximity, thus forming the catalytic site on the surface of domain 1 [5]. The structure of Hbp passenger domain is very similar to *Haemophilus influenzae* IgA protease (IgAP) passenger domain [8]. IgAP is an autotransporter produced by *Haemophilus* and *Neisseria*, which also displays a serine protease domain in the passenger domain. IgAP is involved in the degradation of type 1 immunoglobin A (IgA1), which is found in humans and great apes. Specifically, IgAP cleaves after proline residues located in the hinge region between the Fc and the Fab domains of IgA1. The structural similarity between Hbp and IgAP has led some authors to include IgAP in the SPATE family [8]. However, IgAP, in contrast with SPATEs, does not possess a conserved N-terminal extension, is not cleaved between two asparagines, nor does it possess the FxxEVNNLNK consensus. Additionally, IgAP is processed by an autoproteolytic mechanism that depends on its passenger domain proteolytic activity, rather than upon the β-domain. Also, IgAP does not possess the conserved β-domain sequence found in SPATEs, and is not produced by *Enterobacteriaceae*. Furthermore, whereas IgAP specifically cleaves the hinge region of IgA1, none of the SPATEs for which this substrate has been tested do. Thus, despite structural homology, these enzymes show a clear difference in substrate specificity. The variation in substrate specificity is probably due to the presence of a loop in IgAP, which is absent from SPATEs [5,8,69]. This loop was proposed to act as a lid for the IgAP catalytic site that can only be opened upon binding to the IgA1 Fc region [8]. Also, the catalytic site of Hbp was found to be much more open than IgAP [8,69]. 

**Table 2:** SPATEs substrate specificity. Listed are the substrates for which SPATE activity has been tested.

	Cleaved	Not cleaved
**EatA**	AAPM-*p*NA, AAPL-*p*NA [25]	
**EpeA**	pepsin A, gelatin, mucin [16]	
**EspC**	fodrin [71], hemoglobin [72], pepsin, factor V, spectrin (fodrin) [20,40]	Human IgA1 [71], mucin [40], lysozyme [73]
**EspI**	pepsin A, apolipoprotein A1 [26]	IgA1, hemoglobin, bovine serum albumin, α2-macroglobulin, haptoglobin, thrombin, collagene type 3, trypsin, high density lipoprotein, low density lipoprotein, very low density lipoprotein, trypsin, transferrin, lactoferrin, pepsinogen, gelatin, casein [26]
**EspP**	pepsin A, human coagulation factor V [14], casein [15], apolipoprotein A1 [26], AAPL-*p*NA [40]	human IgA1, bovine serum albumin, α2-macroglobulin, transferrin, lactoferrin, pepsinogen [14], mucin, spectrin (fodrin) [40]
**Hbp**	hemoglobin [5]	albumin, human lactoferrin, human immunoglobulin A1 [5]
**Pet**	casein, gelatin [17], pepsin, human coagulation factor V, spectrin [19,40]	actin [75], mucin [19,40]
**Pic**	gelatin, ovomucin, bovine mucin, murine mucin [18], human spectrin (fodrin), pepsin A, human coagulation factor V [19,40], mucin [19]	casein, IgA, IgM, IgG, hog gastric mucin [18] ovine spectrin [40]
**Sat**	casein [22], factor V, spectrin [40]	IgA1 [22], hemoglobin, mucin, pepsin [40]
**SepA**	FLF-*p*NA, VPF-*p*NA, AAPF-*p*NA, AAPM-*p*NA [70]	IgA1, gelatin [28], angiotensin-I, egg lysozyme [70], fibronectin, mucin, pepsin, factor V, spectrin (fodrin) [40]
**SigA**	casein [29], fodrin [45]	
**Tsh**	mucin, factor V [40]	human and chicken IgA, casein, pepsin A [40,42], spectrin [40]
**Vat**		casein [35]

Although Hbp is the only SPATE for which a passenger domain structure is available, homology modeling of EspP and Pet suggests that these SPATE passenger domains adopt a very similar fold compared with Hbp [5,68]. In particular, the domain 1 of EspP was predicted to adopt an elastase-like fold, suggesting that in all SPATEs, domain 1 adopts a chymotrypsin-like fold [68]. The variation in substrate specificity between SPATEs (Table 2) could then be explained by residue variation in the specificity pocket [5]. Although the basis for SPATEs’ substrate specificity has not been demonstrated, a study with SepA and a collection of synthetic oligopeptides suggests that the length of the substrate is important for proper cleavage. Also, although SepA interacts with other residues from the cleavage site, the residue at position P1 (Phenylalanine in the case of SepA, but Pro for Iga-Pr) is particularly important for cleavage [70]. However, SepA seems to also interact with other residues of the cleavage site [70]. In the same study, the optimum conditions for SepA activity were 37 °C and near neutral pH (7.5) [70]. 

The serine protease activity of SPATE passenger domains is not involved in the processing of the passenger domain from the β-domain and is not related functionally to the proteolytic activity of the β-domain. In contrast to the β-domain proteolytic activity, which is apparently only required for the release of the passenger domain in the extracellular space, the proteolytic activity located in the passenger domain might be important for the virulence of the producing strain. The proteolytic activity of SPATE passenger domains has been tested *in vitro* both on synthetic oligopeptides labeled with paranitroanilide (*p*-AN) or on entire proteins. As can be seen in Table 2, SPATEs are very diverse in their substrate specificity. 

Whether the ability of SPATEs to cleave the proteins tested *in vitro* is relevant to their role *in vivo* is mostly unknown. It has been proposed that the cleavage of mucin might help pathogenic bacteria degrade mucus and thus adhere to the mucosa [18]. The cleavage of human coagulation factor V by EspP has been suggested to be involved in the hemorrhage observed upon infection by EHEC [14]. Finally, fodrin might be the intracellular target of EspC and Pet [40,71]. However, these hypotheses have not been tested and for most SPATEs, the actual *in vivo* target is still unknown. 

### 5.2. Role in Pathogenesis

SPATEs have been divided into two sub-groups based upon phylogenic criteria. One group includes SPATEs that display cytotoxic activity (Pet, EspC, EspP, SigA) and, which probably have intracellular targets. The other group includes SPATEs that do not have demonstrated cytopathic activity on cells, but which might be important in pathogenesis because they affect an extracellular target [31]. Here, we summarize studies addressing the role of SPATEs in the virulence of their parental strain.

EaaA/EaaC: *eaaA* and *eaaC* are two genes that have been identified in the non-pathogenic *E. coli* strain ECOR-9 (this strain was isolated from the feces of a healthy Swedish child). The two genes are located on prophages and are linked to genes encoding non-SPATE, trimeric autotransporters involved in non-immune IgG and IgA binding (*eib* “*E. coli* Ig binding” genes). *eaaA* and *eaaC* are 99.4% identical at the nucleotide level and encode proteins homologous to the SPATE proteins. The actual expression of *eaaA* and *eaaC* in ECOR-9 or the role of these genes has not been tested [27]. Although autotransporters are often referred to as virulence factors, the presence of these genes in a non-pathogenic strain of *E. coli* suggests that SPATE proteins might not all be virulence factors *per se*. Certain SPATEs might be important for colonization of the gastrointestinal tract by commensal strains, but this needs to be tested.

EatA: EatA is produced by enterotoxigenic strains of *E.coli* (ETEC), which are a major cause of diarrhea in developing countries. EatA is 73% identical to SepA and displays similar enterotoxic effects in the rabbit ileal loop model of infection. In a manner that is similar to a *Shigella sepA* mutant [28], at 7 h following infection, a ETEC *eatA* mutant caused less fluid accumulation and mucosal destruction in this model than the wild-type strain would. However, after 16 h of infection, no difference was observed between the mutant and the parental strain (*sepA* effects 16 h after infection were not reported [28]). These results suggest that EatA is not necessary for ETEC virulence, but might accelerate the development of the disease. Whether EatA proteolytic activity is necessary for this effect has not been tested. Indeed, EatA, like other SPATEs, is a serine protease. EatA cleaves the oligopeptide methoxysuccinyl (MeOSuc)-Ala-Ala-pro-Met-*p*-nitroanilide (AAPM-*p*NA: 100% activity) *in vitro*, as well as AAPL-*p*NA (80%), AAPF-*p*NA (10%), VPF-*p*NA (30%) and FLF-*p*NA (10%) [25]. The substrates cleaved by EatA *in vitro* are also substrates for cathepsin G (an antibacterial serine protease secreted by polymorphonuclear leukocytes) and SepA. However, despite a strong homology with EatA, SepA levels of activity on these substrates are different: 50% for AAPM-*p*NA and AAPF-*p*NA: 50%, 100% for VPF-*p*NA, and 80% for FLF-*p*NA [70]. 

EpeA: *epeA* was identified by sequencing the plasmid pO113 transfer region from non-LEE EHEC [16]. EpeA expression was verified and its toxic effect on HeLa cells was tested. In contrast with EspP, Pet, SigA or Sat, no toxicity was observed when purified EpeA was applied on HeLa cells [16]. This is consistent with the split decomposition analysis from Yen *et al.* that shows that EpeA belong to the non-cytotoxic group of SPATEs [31].

EspC: Upon infection, EPEC form characteristic “attaching and effacing” (A/E) lesions on the intestine mucosa. These lesions are characterized by the effacement of microvilli and the formation of an actin pedestal underneath the adherent bacterium. Although the role of these lesions in pathogenesis is not clear, factors involved in their formation are important for virulence. EspC is secreted by EPEC and was initially shown to associate with HeLa cells *in vitro*, suggesting that it might have a role in virulence. However, an *espC* deletion mutant was not different from its isogenic EPEC parent in terms of adherence or invasion of HeLa, HEp-2, or polarized Caco-2 cells. In addition, EspC was shown not to be involved in the formation of A/E lesions: first, the Δ*espC* mutant was not affected in EPEC-mediated epithelial cell signaling, cytoskeletal rearrangements, or Tir-phosphorylation, all of which are necessary for A/E lesion formation [41]. Second, EspC was not detected in several other pathogens, such as *Hafnia alvei* or *Citrobacter freundii*, which also form A/E lesions, indicating that EspC is not required for this phenomenon to occur [20]. EspC also does not appear to be involved in inhibition of complement killing or hemagglutination [20]. However, EspC mediates EPEC lysozyme resistance, by an unknown mechanism independent of its proteolytic activity. This could be important for initial steps of the disease and resistance of the bacterium to non-specific host defense [73]. Also, purified EspC displays enterotoxic activity on rat jejunal tissue mounted in Ussing chambers [33] as well as cytotoxicity on epithelial cells *in vitro* [71]. The effects of purified EspC on epitelial cells (formation of vacuoles, cell contraction, cells detachment and rounding, membrane blebs and cytoskeletal damage) were similar to those observed with another SPATE: Pet. However, compared with Pet, EspC required a longer incubation time with cells and a higher concentration to produce the same effects [71]. Whether the enterotoxic effect of EspC was dependent upon its proteolytic activity was not tested [33]. In contrast, the cytotoxic effect of purified EspC on epithelial cells was dependent on the protease activity of the passenger domain: addition of the protease inhibitor PMSF (phenylmethylsulfonyl fluoride) eliminated all cytotoxic effects, and no cytotoxicity was observed in cells incubated with a EspC mutant defective in protease activity (S256I) [71]. A potential intracellular substrate for EspC is fodrin, a ubiquitous protein involved in actin crosslinking, which is also cleaved by the SPATE Pet. EspC binds and cleaves fodrin *in vitro* and the cleavage sites recognized by EspC in fodrin are different from those recognized by Pet. Also, whereas fodrin cleavage by Pet triggers foldrin redistribution in HeLa cells, such effects were not observed upon incubation of cells withEspC [74]. However, whether EspC actually cleaves fodrin *in vivo* or whether the cytotoxic effects induced by EspC are due to fodrin cleavage has not been determined. It is currently not known if the cleavage of other EspC substrates identified *in vitro* (hemoglobin, mucin *etc.*) is physiologically relevant and has any role in pathogenesis. Finally, EspC cytotoxic effects are strictly dependent upon the protein internalization in the cytoplasm of epithelial cells. Purified EspC can be internalized by pinocytosis in a receptor-independent fashion when added to epithelial cells at high concentration for a long (8 h) incubation time [75]. However, this process is not efficient, nor physiologically relevant, since it does not involve any receptor or intracellular trafficking. In contrast, when EPEC infects cells, EspC is efficiently internalized by a mechanism that depends upon the presence of a functional type III secretion system. Although EspC is secreted by the type V secretion system, it then interacts with components of the EPEC Type III secretion system (EspA, the tip protein) and is internalized by a process dependant upon this system. Interestingly, this internalization can also be triggered by T3SS from other strains, such as EHEC or REPEC. This process is highly specific for EspC since another SPATE (Pic) was not internalized in the same conditions [76]. It is generally assumed that type III secretion systems translocate proteins directly from the cytoplasm of the bacterium to the cytoplasm of eukaryotic cells in one step through a closed conduit. How exactly EspC “hijacks” the type III secretion system from the extracellular medium, where it is secreted, to gain access to the eukaryotic cell cytoplasm is not known. EspC interacts with EspA, the EPEC T3SS tip protein, which is exported by the T3SS and assembled at the tip of the needle following completion of the needle structure. Then, the T3SS interacts with host cells and forms a pore in the host cell membrane through which T3SS effectors can be translocated [77]. Since EspC is the first protein secreted by EPEC upon infection [41], the interaction between EspA and EspC could be taking place while the needle is being assembled, before the needle actually interacts with the cell. If this is the case, EspC would be already localized at the tip of the needle when the EspD/B pore is formed in the host cell membrane. Thus, EspC might use this pore to gain access to the host cell cytoplasm. However, if this is true, EspC might not be able to gain access to the pore once the T3SS apparatus is finalized and a closed channel is formed between the bacterial cytoplasm and the host cell cytoplasm. As a consequence, EspC could only be internalized if synthesized before or during the T3SS needle assembly. Thus, delaying expression of EspC would probably reduce its internalization. In addition, it has been estimated that the pore formed by T3SS in the host membrane is ~25Å wide [78], which is too small to accommodate a folded protein the size of EspC. Thus, EspC would have to be unfolded if it were to use this pore for internalization. Another possibility would be that EspC interaction with EspA is a way for the bacterium to avoid diffusion of EspC in the medium after secretion or to target EspC to the host cell, or both. After reaching the cell membrane via the T3SS, EspC could then be translocated through the host membrane by a T3SS-independent mechanism. For example, EspC could use a “membrane puncturing” mechanism similar to the one used by the T4 phage gp5 needle protein or the *Vibrio cholerae* T6SS VgrG protein: both of these proteins form long β-helices that are able to perforate membranes without the need of a pore or a dedicated translocase [80]. Also, VacA, a non-SPATE autotransporter that folds as a β-helix [7], is able to insert in cell membrane, where it forms pore [81]. Thus, β-helical proteins seem to have a certain ability to insert into membrane, and since EspC, like most autotransporter passenger domains, is predicted to fold as a long β-helix, it might also be able to insert in the membrane independently of the T3SS. However, although EspC might cross the host membrane by itself, the interaction with the T3SS needle might be important for proper orientation of EspC toward the membrane (most likely perpendicular to the plane of the membrane) or to provide the energy required for translocation through the host membrane, or both.

EspI: *espI* was identified as one of the ORFs present in a novel pathogenicity island found in the STEC subgroup that lacks the locus of enterocytes effacement (LEE). Like EspP, EpeA, EspC, Pet, and Pic, purified EspI is able to digest pepsin A1. However, like EpeA, it is unable to elicit toxic activity when applied to Vero cells [26], suggesting that, like EpeA, EspI belongs to the subtype of SPATE that possesses an extracellular target, rather than an intracellular one. This classification of EspI is supported by the phylogenetic studies of Yen *et al*. [31]. Interestingly, EspI was also found to cleave apolipoprotein A-I, a protein present in human serum, but whether this is the actual target of EspI *in vivo* is not known [26].

EspP/PssA: EspP/PssA is produced by EHEC [14,15]. EspP/PssA was shown to induce cytotoxic effects on Vero cells. In a manner similar to EspC and Pet, purified EspP/PssA induced cytoskeletal damage, with loss of stress fiber, disruption of actin cytoskeleton, cell detachment and rounding, and opening of the cell-cell junction [15] Whether EspP/PssA is, like Pet and EspC, internalized by host cells is not known. Also, the role of EspP/PssA proteolytic activity in its cytotoxicity has not been reported, but it is possible that EspP also cleaves fodrin *in vivo*, since the cytopathic effects observed with EspP/PssA are similar to the ones observed with EspC and Pet, both of which target fodrin. In addition, a role for EspP/PssA in intestinal colonization of cattle was suggested by isolation of the *pssA* mutant in a signature-tagged mutagenesis screen [81]. EspP/PssA was also proposed to have a role in *E. coli* O157:H7 colonization of intestinal mucosa and adherence based upon experiments done with an *E. coli* O157:H7 *espP*::kan^R^ mutant, which was shown to be slightly affected in adherence to cultured cattle rectal cells *in vitro* [82]. In a more recent study, a *ΔespP* derivative of the *E. coli* O157:H7 strain EDL933, when compared with the parental strain, was also deficient in adherence to T84 colonic adenocarcinoma cells *in vitro* [83]. However, when EHEC O157:H7 is cured of the plasmid pO157 (which encodes EspP), the pathogenesis in gnotobiotic piglets is not affected [84]. Interestingly, *espP* was also one of the genes isolated in a screen for genes involved in biofilm formation [83]. The role of EspP proteolytic activity in adherence or biofilm formation has not been investigated. Finally, cleavage of coagulation factor V by EspP has been proposed to be involved in the hemorrage observed during EHEC infection; however this has not been proven yet. 

Hbp: Hbp “hemoglobin protease” or “hemoglobin binding protein” was initially identified in an *E. coli* strain (EB1) isolated from a patient presenting a wound infection [21]. Hbp can interact with heme and hemoglobin *in vitro*, but has better affinity for hemoglobin than heme. Hbp can also degrade hemoglobin, by a mechanism dependent upon its serine protease activity. Although it has been proposed that the Hbp heme-binding domain could be located between residues 608 and 644, this has not been tested experimentally [85]. The hemoglobin-binding domain is not known either. The Hbp passenger domain crystal structure has been resolved, but only in the apo form [5]. Thus, no information is currently available on the heme binding-site location or on potential conformational change(s) occurring in the protein after heme or hemoglobin binding [5]. Because Hbp is secreted in the extracellular milieu and binds heme, it was proposed to be part of an iron acquisition system. In gram-negative bacteria, heme-acquisition occurs by two distinct mechanisms. In one mechanism, bacteria directly bind heme or the heme source through a membrane receptor and then transfer the heme into the bacterial cytoplasm. In the second mechanism, the bacterium secretes a heme-binding protein (hemophore) into the milieu. This hemophore scavenges heme from hemoproteins and then shuttles heme back to the bacteria, where it interacts with a specific receptor. Although Hbp is similar to hemophores because it is secreted and binds heme, it is also a protease, which degrades hemoglobin. Thus, Hbp could participate in heme-acquisition by either of the two mechanisms. In one case, Hbp-mediated hemoglobin degradation could release free heme molecules into the medium. These molecules would subsequently be taken up by a bacterial heme-receptor such, as *E. coli* ChuA. However, because Hbp binds heme with high affinity, the release of free heme in the media would probably need to occur after all Hbp molecules have been converted to holo-Hbp. Assuming that heme-binding does not affect Hbp proteolytic activity (which to our knowledge has not been tested), cleavage of hemoglobin by holo-Hbp would release free heme in the environment (because no empty heme binding pocket is available on Hbp). Because hemoglobin triggers release of Hbp from hemin-agarose [5], it is also possible that holo-Hbp releases the bound heme molecule when interacting with a new molecule of hemoglobin. Alternatively, Hbp could act as a hemophore: in which case it can bind hemoglobin, degrade it, and bind the released heme. Then, holo-Hbp would need to deliver the bound heme to the bacterium. However, no receptor for Hbp on the bacterial outer membrane has been identified so far. The Hbp beta-barrel, which would be left in the bacterial membrane after cleavage and release of the passenger, is probably not this receptor since Hbp has been shown to deliver heme to *Bacteroides fragilis*, which does not express Hbp [85]. The ability of *B. fragilis* to capture heme from *E.coli* Hbp is probably one of the reasons for the synergy of abscess formation in intra-abdominal infections caused by *E. coli* and *B. fragilis*. Indeed, Hbp is essential for abscess formation in the context of coinfection by *E. coli* and *B. fragilis*. Immunization of mice with Hbp prevented such abscess formation [85].

Whether Hbp acts as a hemophore or only as a hemoglobin-protease has not been clearly established yet. However experiments performed with *B. fragilis* suggest a role for Hbp as a hemophore. Growth of *B. fragilis* can be made heme-dependent in certain medium [85]. In these conditions, growth is stimulated upon addition of heme or purified holo-Hbp to the medium. Apo-Hbp, in contrast, does not promote growth. The effect observed with holo-Hbp was not due to the release of free heme from Holo-Hbp, because (1) twice as much free heme was required to give the same effect as holo-Hbp (heme and Hbp bind in a 1:1 ratio) and (2) antibodies directed against Hbp abolished holo-Hbp growth induction [85]. These results suggest that *B. fragilis* has a specific receptor for holo-Hbp and can transfer heme from holo-Hbp. Whether *E. coli* EB1 (the strain in which Hbp was initially identified) also has such a receptor remains to be determined.

Finally, the last possibility would be that holo-Hbp transfers heme to another hemophore with higher affinity for heme and that this second hemophore then shuttles the heme back to the bacteria. However, such a mechanism has never been described and we are not aware of any other heme-binding protein secreted by *E.coli* EB1. 

The ability to cleave hemoglobin has been tested only for a few of the other SPATEs; whereas Sat and EspI do not process hemoglobin [26,40], EspC and Pic do [67], but in these cases the binding to heme has not been tested. Thus, whether EspC and Pic could also be part of iron acquisition systems is not known. 

Finally, Hbp and Tsh only differ by two residues (Q209K and A842T). Tsh, like Hbp was able to induce abscess formation in a mouse model of coinfection with *B. fragilis*, suggesting that it also has the ability to cleave hemoglobin and/or bind heme. However no report of such activity is available [81]. However, Hbp, in contrast with Tsh, does not show any mannose-resistant hemagglutination activity, at least not in the conditions tested [21], suggesting that the two divergent residues are important for this particular activity, but not for abscess formation.

Pet: Pet was the first autotransporter for which an enterotoxic activity was reported [17]. Specifically, Pet shows enterotoxic activity on rat jejunal tissue mounted in Ussing chambers [17,86]. Pet also causes cytotoxic effects on these same cells: tissue damage, inflammation, and mucus secretion were observed [86]. Furthermore, Pet was found to induce cytopathic effects on HEp-2 and HT29 C1 cells. Both enterotoxic and cytotoxic activity are dependent upon the proteolytic activity of the Pet passenger domain, since a mutant in the catalytic serine is not toxic and the use of serine protease inhibitors abolishes these toxic effects [82]. In a manner similar to EspC, Pet toxic effects require internalization of the protein into host cells. However, in contrast with EspC, the internalization of Pet occurs by Clathrin-dependent endocytosis [87]. After endocytosis, Pet is trafficked to the endoplasmic reticulum and back to the cytosol [88]. Once in the cytoplasm, Pet cleaves the same substrate as EspC: fodrin (α-spectrin). This cleavage induces a redistribution of fodrin and the cytopathic effects observed.

Pic: Pic is produced by *Shigella flexneri*, EAEC, and UPEC [18,19]. Pic was shown to mediate serum resistance by a mechanism dependent upon its proteolytic activity. Although the exact mechanism of this serum resistance is not known, it is probable that Pic degrades one of the components of the complement classical pathway of activation [18]. Pic also mediates species-specific hemagglutination (weak hemagglutination was observed for rat, pig, rabbit, horse, and sheep red blood cells, but none was observed with human or chicken red blood cells)[18]. No effect of Pic as a cytotoxin was detected [86]. However, it was shown that Pic can cleave fodrin, the intracellular target of EspC and Pet cytotoxins [19]. In UPEC, the wild-type strain does not have an advantage over a *pic* mutant in colonization of the urethra, bladder, or kidney [89]. In contrast, in streptomycin-treated mice, EAEC strain 042 was more efficient at colonizing the gastrointestinal tract than either a *pic* mutant or a strain expressing Pic with no proteolytic activity (042PicS258A) [90]. However, the difference in colonization did not correlate with a variation in adherence or growth *ex vivo*, thus suggesting another role for Pic. Interestingly, it was shown that 042PicS258A is affected in its ability to grow in the presence of mucin *in vitro*. It was therefore proposed that Pic might have a nutritional role in colonization. Specifically, Pic may allow bacteria to use mucus or mucin as a nutrients source when other sources are not available [90].

Sat: Sat is produced by UPEC, a leading cause of urinary tract infection. Sat is cytotoxic on VERO kidney cells, HK-2 human bladder, and HEp-2 cell lines. Like Pet or EspC, Sat contact with culture cells leads to cell elongation and detachment from their support [22]. Sat also causes vacuolation of bladder and kidney cells [91]. This activity is dependant upon the protease activity of Sat and upon its internalization in host cells [92]. The ability of Sat to cleave fodrin *in vitro* suggests that, like EspC and Pet, Sat might target fodrin *in vivo*. No difference between wild-type and *sat* mutant were observed in a short-term assessment of colonization in a mouse model of ascending urinary tract infection [22]. However, less cellular damages were observed in the kidneys of mice infected with a *sat* mutant than in those infected with the wild-type strain, thus suggesting that Sat acts mainly as a toxin [92]. In contrast with Tsh, Sat did not exhibit any hemagglutinin activity [22]. The *sat* gene is also found in *Shigella* and DAEC. In DAEC, Sat was shown to promote lesions in tight junctions between intestinal epithelial cells. This activity was dependent upon the proteolytic activity of Sat, but the exact target in unknown [93].

SepA: SepA is one of the SPATEs produced by *Shigella flexneri*, the agent of Shigellosis, a disease in which bacteria invade the colonic mucosa and trigger a strong inflammatory response. SepA is 72% identical to EatA, an enterotoxin produced by ETEC (see above). SepA is not involved in bacterial entry in HeLa cells, plaque formation on Caco-2 cells, or dissemination between cells [28]. However, a *sepA* mutant is attenuated in the rabbit model of ligated ileal loop. Infection with this mutant indeed causes reduced fluid accumulation, reduced mucosal atrophy and decreased tissue inflammation compared with the wild-type strain 8h after inoculation [28]. However, the effect of SepA in the rabbit ileal loop model was not tested at later time points, and in the case of EatA, although similar, toxic effects were seen 7 h after infection, after 16 h, no difference between the mutant and the wild-type subsisted [25]. Thus, it is possible that SepA, like EatA, is not essential for the establishment of the disease but increases the disease’s rate of progression. The role of SepA proteolytic activity in its enterotoxic activity was not tested [28]. SepA substrate specificity, however, was tested *in vitro* on synthetic peptides, and showed similarity to cathepsin G. However, in contrast with cathepsin G, SepA was not able to activate platelets, cleave thrombin receptors, fibronectin, collagen or angiotensin I. This indicates that despite *in vitro* similarity, the SepA *in vivo* target is probably not one of the cathepsin G substrates [70]. As discussed earlier, SepA substrate specificity is also similar to EatA, but the level of activity on the substrates differs between the two SPATEs, suggesting that they probably do not share the same *in vivo* target either [25,70].

SigA: SigA is produced by *Shigella flexneri*. A culture supernatant containing SigA caused damage to HEp-2 cell culture (cell rounding and detachment). However, the effects were less pronounced than the similar effects seen for Pet. Addition of a protease inhibitor abolishes toxicity, indicating that the damage is caused by the ability of SigA to cleave a host target [29]. Indeed, SigA, like Pet and EspC, was shown to degrade fodrin *in vitro* and *in situ* [45]. The cleavage of fodrin *in situ* by SigA causes its redistribution within the cell [45]. In contrast with Pet and EspC, the mechanism of entry of SigA in target cells and the trafficking route following entry has not been determined.

Tsh: The Tsh-encoding gene was initially identified as a gene from avian pathogenic *E. coli* (APEC) strain χ7122. The gene confers hemagglutination properties to *E. coli* K12 [23]. APECs are pathogenic strains of *E. coli* that cause extraintestinal infection in poultry. APEC infections affect the respiratory tract, but can also become generalized and cause fatal septicemia in animals. Tsh hemagglutination activity was not abolished in a strain expressing a mutant where the Tsh catalytic serine was changed to threonine or alanine, suggesting that the hemagglutinin activity is independent from the proteolytic activity [42] Interestingly, Tsh-producing strain χ7122 was, like the Hbp-producing strain, able to induce a synergistic interaction with *B. fragilis* in a mouse model of intra-abdominal abscess formation [85]. This suggests that Tsh, like Hbp, can degrade hemoglobin and transfer heme obtained from hemoglobin degradation to *B.fragilis*. Indeed, Tsh was shown to bind hemoglobin [94]. Tsh also binds collagen IV and fibronectin, suggesting that it might act as an adhesin [94]. Tsh, in contrast with Pic, does not mediate serum resistance [32], but was proposed to be important for the colonization of the chicken air sac. Indeed, a *tsh* knockout mutant of *E.coli* χ7122 causes fewer and less pronounced lesions in these organs in comparison with the parental strain [32]. Tsh is also expressed by UPEC, but the role of Tsh in urinary tract infections has not been investigated [89].

Vat: APEC causes extraintestinal infection in poultry, such as respiratory diseases, cellulites, or septicemia. Vat, like Sat and EspC, has vacuolating activity: it induces the formation of intracellular vacuoles in cell culture. No vacuolating activity was reported for Tsh despite being 75% identical to Vat. A *vat* mutant was attenuated in a cellulite model of infection in chicken [35], suggesting a role for this protein in infection. Whether this activity is, like for Sat, linked to its protease activity is not known. So far no substrates have been identified for Vat. Interestingly, Vat is the only SPATE for which the serine protease consensus is not perfectly conserved (ATSGSP instead of GDSGSP). Thus, the proteolytic activity and substrate specificity of this particular SPATE would be particularly interesting to look at. 

## 6. Conclusions

Autotransporters are an expanding family of secreted proteins from gram-negative bacteria. Over the past two decades, numerous studies have focused on elucidating the secretion mechanism of these proteins. However, so far, the mechanism by which these proteins cross the outer membrane remains controversial. The discrepancy of data obtained between different studies actually suggests that different autotransporters might use different secretion mechanisms. It is already known that these proteins show variations in their biogenesis (cleavage mechanism, chaperone requirement) and function (protease, adhesins). Thus, differences in secretion would not be that surprising. The AT variety is particularly striking in the SPATE family of autotransporters. Although SPATEs are much more conserved than other autotransporters, discrepancies are also observed between studies addressing their secretion mechanism. Whether this is due to variation in experimental conditions or not will need to be addressed in the future. In addition, SPATEs show huge diversity in function. All these proteins are serine proteases, and probably adopt a similar fold, but still, they do not share the same targets. In addition, their effect on cells varies: some SPATEs are cytotoxins, while others are not. The mechanism SPATE cytotoxins use to enter host cells also varies. Their trafficking routes inside these cells also vary. Still, all these proteins have effects *in vitro* that suggest they are somehow involved in the pathogenesis of their parental strain. Currently, the determination of the role of SPATEs in pathogenesis is limited by the lack of small-animal models of infection. Hopefully, the development of new animal models will permit precise understandings of the roles these proteins play in establishment of disease. Meanwhile, a lot of questions regarding the evolution and biogenesis of SPATEs remain to be answer, including: what is the common ancestor of SPATEs? What exactly is the role of the N-terminal extension, and why is it so conserved in SPATEs? What is the role of the autocleavage? Why do SPATEs not use their serine protease activity to cleave their passenger domains, like other serine protease autotransporters? What is the molecular basis for substrate recognition and specificity? How is SPATE expression regulated? What differentiates SPATEs that are internalized in host cells from SPATEs that are not?

Considering that most SPATEs are proven or putative virulence factors, understanding the details of their biogenesis and function would be particularly useful in designing new antimicrobial therapies.

## Acknowledgments

We are grateful to Travis Barnard and Todd Holyoak for their help in constructing Figure 1, and to Frank Shewmaker for valuable comments on the manuscript.
